# ICTV Virus Taxonomy Profile: *Tosoviridae* 2023

**DOI:** 10.1099/jgv.0.001908

**Published:** 2023-10-24

**Authors:** Yuri I. Wolf, Mart Krupovic, Jens H. Kuhn, Eugene V. Koonin

**Affiliations:** ^1^​ National Center for Biotechnology Information, National Library of Medicine, National Institutes of Health, Bethesda, MD 20894, USA; ^2^​ Institut Pasteur, Université Paris Cité, Archaeal Virology Unit, Paris 75015, France; ^3^​ Integrated Research Facility at Fort Detrick, National Institute of Allergy and Infectious Diseases, National Institutes of Health, Fort Detrick, Frederick, MD 21702, USA

**Keywords:** ICTV Report, Taxonomy, *Tosoviridae*, fraservirus, turtle fraservirus 1

## Abstract

*Tosoviridae* is a family of negative-sense RNA viruses with genomes totaling about 12.3 kb that have been found in turtles. The tosovirid genome consists of two segments, each with two open reading frames (ORFs) in ambisense orientation. The small (S) segment encodes a nucleoprotein (NP) and a glycoprotein precursor (GPC); the large (L) segment encodes an L protein containing an RNA-directed RNA polymerase (RdRP) domain and a zinc-binding (Z) protein. This is a summary of the International Committee on Taxonomy of Viruses (ICTV) Report on the family *Tosoviridae*, which is available at ictv.global/report/tosoviridae.

## Virion

Tosovirids produce enveloped round or slightly oval particles 110–125 nm in diameter [1, 2] (Table 1).

**Table 1.** Characteristics of members of the family *Tosoviridae*

Example	turtle fraservirus 1 (S segment: MZ458542; L segment: MZ458543), species *Fraservirus testudinis*, genus *Fraservirus*
Virion	Enveloped, round or slightly oval; 110–125 nm
Genome	About 12.3 kb of bisegmented negative-sense RNA
Replication	Unknown
Translation	Unknown
Host range	Emydid and trionychid turtles
Taxonomy	Realm *Riboviria*, kingdom *Orthornavirae*, phylum *Negarnaviricota*; the family includes the genus *Fraservirus* and one species.

## Genome

**Fig. 1.**
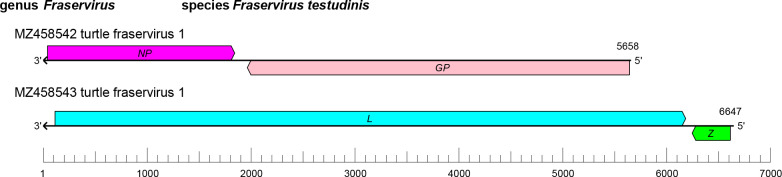
Genome organisation of turtle fraservirus 1. ORFs are coloured according to the predicted protein function (*GP*, glycoprotein precursor gene; *NP*, nucleoprotein gene; *L*, large protein gene encoding an RNA-directed RNA polymerase [RdRP] domain; *Z*, zinc-binding protein gene).

The tosovirid genome comprises two segments (S and L) of linear negative-sense RNA with a total length of about 12.3 kb (S segment: about 5.7 kb; L segment: 6.6 kb). Each segment contains two ORFs in ambisense orientation (Fig. 1). The S segment encodes an NP and a GPC; the L segment encodes an L protein with an RdRP domain, and a Z protein [1]. Superficially, this organization is reminiscent of that of arenavirid genomes [3].

## Replication

Unknown.

## Pathogenicity

Turtle fraservirus 1 is highly virulent in emydid and trionychid turtles [1].

## Taxonomy

**Fig. 2.**
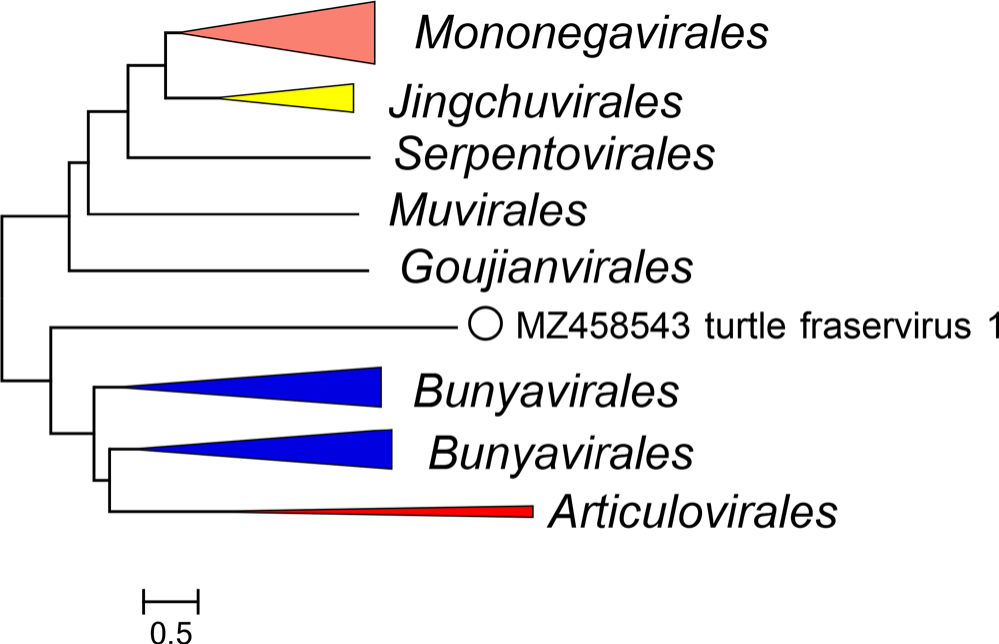
Phylogenetic relationships of turtle fraservirus 1. A phylogenetic tree was reconstructed for an alignment of consensus sequences of the RdRP core domains of the ICTV Virus Metadata Resource (VMR) set of *Negarnaviricota* [5] using the FastTree programme. Branches are collapsed to order level. For full details see *Tosoviridae* ICTV Report.

Current taxonomy: ictv.global/taxonomy. The family *Tosoviridae* includes the genus *Fraservirus* and the species *Fraservirus testudinis* for viruses that infect turtles. Tosovirids are most closely related to, but distinct from, viruses in the subphyla *Haploviricotina* and *Polyploviricotina* [1, 4] (Fig. 2).

## Resources

Full ICTV Report on the family *Tosoviridae*: www.ictv.global/report/tosoviridae
